# The WID-CIN test identifies women with, and at risk of, cervical intraepithelial neoplasia grade 3 and invasive cervical cancer

**DOI:** 10.1186/s13073-022-01116-9

**Published:** 2022-10-19

**Authors:** James E. Barrett, Karin Sundström, Allison Jones, Iona Evans, Jiangrong Wang, Chiara Herzog, Joakim Dillner, Martin Widschwendter

**Affiliations:** 1 European Translational Oncology Prevention and Screening (EUTOPS) Institute, Universität Innsbruck, Milser Straße 10, 6060 Hall in Tirol, Austria; 2 Research Institute for Biomedical Aging Research, Universität Innsbruck, 6020 Innsbruck, Austria; 3 Department of Women’s Cancer, UCL EGA Institute for Women’s Health, University College London, 74 Huntley Street, London, WC1E 6AU UK; 4 Department of Laboratory Medicine, Division of Pathology, Karolinska Institutet, Stockholm, Sweden; 5 Medical Diagnostics Karolinska, Karolinska University Hospital, Stockholm, Sweden

**Keywords:** DNA methylation, Cervical cancer screening, Diagnostics, Liquid-based cytology

## Abstract

**Background:**

Cervical screening is transitioning from primary cytology to primary human papillomavirus (HPV) testing. HPV testing is highly sensitive but there is currently no high-specificity triage method for colposcopy referral to detect cervical intraepithelial neoplasia grade 3 or above (CIN3+) in women positive for high-risk (hr) HPV subtypes. An objective, automatable test that could accurately perform triage, independently of sample heterogeneity and age, is urgently required.

**Methods:**

We analyzed DNA methylation at ~850,000 CpG sites across the genome in a total of 1254 cervical liquid-based cytology (LBC) samples from cases of screen-detected histologically verified CIN1-3+ (98% hrHPV-positive) and population-based control women free from any cervical disease (100% hrHPV-positive). Samples were provided by a state-of-the-art population-based cohort biobank and consisted of (i) a discovery set of 170 CIN3+ cases and 202 hrHPV-positive/cytology-negative controls; (ii) a diagnostic validation set of 87 CIN3+, 90 CIN2, 166 CIN1, and 111 hrHPV-positive/cytology-negative controls; and (iii) a predictive validation set of 428 cytology-negative samples (418 hrHPV-positive) of which 210 were diagnosed with CIN3+ in the upcoming 1–4 years and 218 remained disease-free.

**Results:**

We developed the WID-CIN (Women’s cancer risk IDentification-Cervical Intraepithelial Neoplasia) test, a DNA methylation signature consisting of 5000 CpG sites. The receiver operating characteristic area under the curve (AUC) in the independent diagnostic validation set was 0.92 (95% CI 0.88–0.96). At 75% specificity (≤CIN1), the overall sensitivity to detect CIN3+ is 89.7% (83.3–96.1) in all and 92.7% (85.9–99.6) and 65.6% (49.2–82.1) in women aged ≥30 and <30. In hrHPV-positive/cytology-negative samples in the predictive validation set, the WID-CIN detected 54.8% (48.0–61.5) cases developing 1–4 years after sample donation in all ages or 56.9% (47.6–66.2) and 53.5% (43.7–63.2) in ≥30 and <30-year-old women, at a specificity of 75%.

**Conclusions:**

The WID-CIN test identifies the vast majority of hrHPV-positive women with current CIN3+ lesions. In the absence of cytologic abnormalities, a positive WID-CIN test result is likely to indicate a significantly increased risk of developing CIN3+ in the near future.

**Supplementary Information:**

The online version contains supplementary material available at 10.1186/s13073-022-01116-9.

## Background

Cervical cancer screening has been the most successful personalized cancer prevention strategy to date [1]; the screening aims to identify women with a pre-invasive lesion, which is then surgically excised.

At this point in time, the majority of countries are changing screening from cytology to human papillomavirus (HPV) testing as the primary screen and utilizing cytology to triage high-risk HPV-positive (hrHPV-pos) women for colposcopic assessment [2]. However, several challenges remain for hrHPV-based screening: hrHPV is highly prevalent in cytology-negative women at up to 24% depending on age and country [3], and even in HPV-vaccinated women, the prevalence of HPV infection is approximately 5% [4]. Cytology (Cyt), which is currently used to triage hrHPV-pos women, was recently estimated to have a sensitivity of 52% and a specificity of 75% for the detection of cervical intraepithelial neoplasia grade 3 or above (CIN3+) [5]. The participation rates in cervical screening amongst European women vary between 40.5 and 81.4% and efforts to increase participation to ≥85% are essential. A recent meta-analysis indicated that self-sampling has a consistently higher acceptance over clinician sampling and this might be one avenue forward to reach more women [6]. HPV testing shows comparable results in self- versus clinician-collected samples [7], but the fact that less than 60% of women who provide a self-collected sample show compliance with follow-up recommendations [8–10] indicates that a test other than cytology (which cannot be carried out reliably on self-collected samples) to triage women based on the same self-collected sample which tested hrHPV-pos should be highly beneficial to reduce loss-to-triage-follow-up.

We [11, 12], along with others (reviewed in [13]), have shown the feasibility of utilizing DNA methylation (DNAme) markers to identify women with pre-invasive or invasive cancers. Recently, Kelly et al. [13] published a comprehensive meta-analysis of the performance of DNAme in cervical samples in women with CIN2+ (cervical intraepithelial neoplasia grade 2 or above) and CIN3+ (cervical intraepithelial neoplasia grade 3 or above); a total of 43 studies provided data on human genes (*CADM1*, *MAL*, *MIR-124-2*, *FAM19A4*, *POU4F3*, *EPB41L3*, *PAX1*, *SOX1*) and HPV16 (L1/L2). The majority of studies (81%) evaluated methylation assays following a hrHPV-pos or Cyt-pos result. The number of samples studied ranged from 33 to 1493. Among those 18 studies, which reported the median age and the age range, in 100% of these studies, the median age was > 30 years, and in 14/18 (78%), the median age was ≥ 35 years. The pooled sensitivity and specificity estimates for CIN3+ were 70.5% (95% CI: 64.8–75.6) and 74.7% (95% CI: 70.8–78.1). When restricting to studies allowing standardization of specificity at 70%, the pooled sensitivity for CIN3+ was 71.1% (95% CI: 65.7–76.0). At a set specificity of 50%, the pooled sensitivity for CIN3+ was 82.3% (95% CI: 77.8–86.1).

The clinical use of DNAme markers to identify women at high risk for CIN3+ has been hindered by several factors:

(i) A suboptimal sensitivity in detecting CIN 3+, particularly in young women below 30 years who have a substantially higher prevalence of hrHPV [3] (and for whom cervical screening is recommended [14]) and thus have an increased need for high-performance triage testing. For instance, the *GynTect* test (which utilizes DNA methylation of six genes) has a sensitivity for CIN3 at 35% in <30-year-old and 76% in ≥30-year-old women [15] and the sensitivity for detecting CIN3+ using the *QIAsure* test (which uses methylation of two genes) is 37.5% in <30-year-old [16] and 78.6% in ≥29-year-old women [17]. Overall, DNAme assays were less sensitive for CIN3+ detection compared to cytology ASCUS+ (atypical squamous cells of undetermined significance positive) (DNA methylation versus ASCUS+: relative sensitivity = 0.87, 95% CI: 0.65–1.17) [13].
(ii) Although the relative specificity of DNAme markers is slightly better than Cyt-pos (DNA methylation versus ASCUS+: relative specificity = 1.37, 95% CI: 1.02–1.85), these assessments have been done almost exclusively on women ≥30 years [13].
(iii) A lack of data prevents judgment as to whether a DNAme marker or marker panel is capable of identifying hrHPV-pos women, which, despite being Cyt-neg at the time of assessment, go on to develop CIN3+ in succeeding years. The only data available (albeit not for CIN3+) are provided by De Strooper et al. demonstrating that the combination of *FAM19A4/mir124-2* DNAme allowed risk prediction for hrHPV-pos/Cyt-neg women to develop an invasive cancer in the future with a sensitivity and specificity of 47% and 75%, respectively [18].
(iv) We have recently shown that among women who had been vaccinated before the age of 17 years the cervical cancer incidence rate ratio is 0.12 (95% CI, 0.00 to 0.34) [19]. The cost-effectiveness benefit/harm ratio of screening in populations with a high vaccination rate will decrease unless the same principle (i.e., epigenome-wide DNAme analysis in a cervical sample) can be utilized to detect or predict the risk for other cancers, in particular women-specific cancers.

In order to diagnose and predict women with cervical (pre) cancer, here, we assessed DNAme at ~850,000 CpGs in cervical liquid-based cytology samples utilizing a cohort-based nested case-control setting and developed a DNAme signature (called Women’s cancer risk IDentification CIN test, WID-CIN test). The WID-CIN test was validated in two independent sets to assess the potential of the test to both detect prevalent and predict incident CIN3+ in hrHPV-pos women.

## Methods

### Cervical liquid-based cytology sample collection

All cervical liquid-based cytology samples processed in the capital region of Stockholm in Sweden are biobanked through a state-of-the-art platform at the Karolinska University Laboratory, Karolinska University Hospital, as previously described [20]. Since the year 2013, virtually 100% of the ~150,000 liquid-based cytology (LBC) samples per year are compacted and stored in a 600-μl, 96-well plate format at −27°C. This allows for the preservation of intact cells and analysis of DNA, RNA, and protein content, among others. The biobank is linked to the Swedish health register infrastructure for cytology/HPV results, histopathology test, and results, as well as cervical cancer diagnoses, through the individually unique personal identification number (PIN) [21].

We defined a cohort of women resident in Stockholm, participating in cervical screening, or clinically indicated testing during the years 2013–2016, and have screening sample(s) stored in the biobank (404,434 women). We linked them to the National Cancer Register at the Swedish National Board of Health and Welfare, and the Swedish National Cervical Screening Registry, to identify all cases of CIN3/adenocarcinoma in situ (AIS) or invasive cervical cancer (CIN3+) diagnosed during 2013–2017. Ethical approval was granted by the Karolinska Ethical Committee (Dnr 2014/1242-31/4).

The experimental design is shown in Table 1. In the discovery and validation sets for CIN3+ diagnosis, all screening-derived samples that were cytology-positive during 1–90 days prior to CIN3+ diagnoses in 2013–2015 were defined as cases. As part of the population was randomized to primary HPV screening in Stockholm during 2014–2016 [22], controls were randomly selected from samples that were hrHPV-pos and Cyt-neg in women having no historical cervical lesions, frequency matched 1:1 on age group and calendar year of samples. Cases and controls were then randomly divided into discovery and validation sets. We also identified samples during 1–90 days prior to histologically diagnosed CIN1 and CIN2 with similar age distribution, to assess the discrimination ability to exclude low-risk lesions. In the predictive validation set for CIN3+ prediction, all cervical samples that were hrHPV-pos and Cyt-neg during 1–4 years prior to CIN3+ diagnoses in 2015–2017 were defined as cases. Random hrHPV-pos and Cyt-neg samples of women who did not have CIN3+ diagnosis in subsequent 1–4 years were selected as controls, frequency matched 1:1 on age group, calendar year, and type of samples (screening or clinically indicated). All samples, which did not have HPV results on record, were put through high-performance HPV testing on the cobas 4800 assay [23], and 10 CIN3+ cases subsequently tested negative for hrHPV.

**Table 1:** Experimental design. Pathological diagnosis included cervical intraepithelial neoplasia (CIN), invasive cancer, and adenocarcinoma in situ (AIS)

**Set**	**Type**	**Cytology-pos**	**Cytology-neg**
**Discovery set**^a^			
n		170	202
Mean age (range)		32.5 (23–62)	34.7 (23–60)
Pathological diagnosis	*Invasive cancer*	6 (3%)	-
	*AIS*	7 (4%)	-
	*CIN3*	157 (93%)	-
**Diagnostic validation set**^b^			
n		343	111
Mean age (range)		33.1 (23–53)	35.2 (23–60)
Pathological diagnosis	*Invasive cancer*	3 (1%)	-
	*CIN3*	84 (25%)	-
	*CIN2*	90 (26%)	-
	*CIN1*	166 (48%)	-
**Predictive validation set**			
n		210	218
Mean age (range)		31.7 (19–50)	31.8 (21–49)
Pathological diagnosis 1–4 years later	*CIN3+*	210 (100%)	-
	*No CIN3+*	-	218 (100%)

^a,b^All cytology-positive and cytology-negative samples were hrHPV-positive apart from 5 hrHPV-negative samples and 1 unknown in the discovery set and 5 hrHPV-negative samples in the diagnostic validation set. In the predictive validation set, 10 samples (out of 428) were hrHPV-negative

To maximize DNA content, we were blinded to case-control status and visually screened all eligible vials of biobanked samples to ensure that a visible cell pellet was present. Approximately 1/3 of samples had such a pellet that was independent of case-control or CIN3/ICC status. We subsequently aliquoted 100 μl from each sample for UCL to perform methylation analyses.

In summary, the three sets consisted of the following samples (Table 1):

(i) Discovery set: 170 and 202 CIN3+ cases and hrHPV-positive/cytology-negative controls, respectively
(ii) Diagnostic validation set: 87, 90, 166, and 111 CIN3+, CIN2, CIN1 cases, and hrHPV-positive/cytology-negative controls, respectively
(iii) Predictive validation set: 428 cytology-negative samples (418 were hrHPV-positive; 10 were hrHPV-negative) of which 210 were diagnosed with CIN3+ in the upcoming 1–4 years and 218 remained disease-free

### Sample processing and DNA extraction

Six hundred fifty-microliters of PBS was added to each 100-μl cervical LBC sample received from the Karolinska University Laboratory biobank and centrifuged for 15 min at 4600 rpm. The supernatant was carefully removed and the pellet was washed with a further 750-μl PBS. The samples were then vortexed and centrifuged again for 15 min at 4600 rpm. After careful removal of the second PBS wash, the samples were re-suspended in lysis buffer from the Nucleo-Mag Blood 200-μl kit (Macherey Nagel, cat #744501.4) which was used in conjunction with the Hamilton Star liquid handling platform for high-throughput DNA extraction. DNA concentration and quality absorbance ratios were measured using Nanodrop-8000, Thermoscientific Inc. Extracted DNA was stored at −80°C until further analysis.

### DNA methylation array analysis

Cervical samples were normalized to 10–25 ng/μl and 200–500 ng total DNA was bisulfite modified using the EZ-96 DNA Methylation-Lightning kit (Zymo Research Corp, cat #D5047) on the Hamilton Star Liquid handling platform. Eight microliters of modified DNA was subjected to methylation analysis on the Illumina InfiniumMethylation EPIC BeadChip (Illumina, CA, USA) at UCL Genomics according to the manufacturer’s standard protocol.

### Methylation analysis

All methylation microarray data were processed through the same standardized pipeline. Raw data was loaded using the R package minfi [24]. Any samples with median methylated and unmethylated intensities <9.5 were removed. Any probes with a detection *p*-value >0.01 were regarded as failed. Any samples with >10% failed probes, and any probes with >10% failure rate were removed from the dataset. Beta values from failed probes (approximately 0.001% of the dataset) were imputed using the impute.knn function as part of the impute R package [25].

Non-CpG probes (2932), SNP-related probes as identified by Zhou et al. [26] (82,108), and chrY probes were removed from the dataset. An additional 6102 previously identified probes that followed a trimodal methylation pattern characteristic of an underlying SNP were removed. Background intensity correction and dye bias correction were performed using the minfi single sample preprocessNoob function. Probe bias correction was performed using the beta mixture quantile normalization (BMIQ) algorithm [27].

The fraction of immune cell contamination, and the relative proportions of different immune cell subtypes in each sample, were estimated using the EpiDISH algorithm [28] using the epithelial, fibroblast, and immune cell reference dataset. The top 1000 most variable probes (ranked by standard deviation) were used in a principal component analysis. Statistical tests were performed in order to identify any anomalous associations between plate, sentrix position, date of array processing, date of DNA creation, study center, immune contamination fraction, age, type (case versus control), and the top ten principal components. No anomalous associations were found.

### Statistical analyses for classifier development

Contamination by immune cells presented a challenge with respect to the identification of differentially methylated positions (DMPs) as differential methylation that occurred solely in epithelial cells was diminished in samples with a high proportion of immune cells (IC) and vice versa. In order to overcome this (as previously described [29]), we linearly regressed the beta values on IC for each CpG site, the linear models being fitted to cases and controls separately. The intercept points at IC = 0 were used as estimates of mean beta values in cases and controls in a pure epithelial cell population. The difference between these intercept points provided a delta-beta estimate in epithelial cells. The difference between intercept points at IC = 1 provided immune cell delta-beta estimates. *p* values for differentially methylated positions were adjusted using Holm multiple testing correction (<0.05).

The R package glmnet [30] was used to train classifiers with a mixing parameter value of alpha = 0 (ridge penalty) and alpha = 1 (lasso penalty) with binomial response type as previously described [29]. Data from the discovery set were used to fit the classifiers. A ranked list of CpGs was generated by taking the CpG with the largest epithelial delta-beta, followed by the CpG with the largest immune delta-beta, followed by the next largest epithelial delta-beta, and so forth (any duplicates were removed). The top *n* CpGs from the list of ranked CpGs were used as inputs to the classifier. Tenfold cross-validation was used inside the training set by the cv.glmnet function in order to determine the optimal value of the regularization parameter lambda. The receiver operating characteristic area under the curve (AUC) was used as a metric of classifier performance. Out-of-bag AUC estimates (based on the cross-validation folds that were not used for training the classifier) were as a function of *n*, the number of CpGs used as inputs during training. The maximum value of *n* was 10,000.

The optimal classifier was selected based on the highest out-of-bag AUC obtained on the discovery set. Once the classifier was finalized, it was then applied to the validation datasets. Denoting the top *n* CpGs as *β*_1_, …, *β*_*n*_ and the regression coefficients from the trained classifier as *w*_1_, …, *w*_*n*_ then WID-CIN index = $\sum_{i=1}^n\left({w}_i{\beta}_i-\mu \right)/\sigma$ where *μ* and *σ* are defined as the mean and standard deviation of the quantity $\sum_{i=1}^n{w}_i{\beta}_i$ in the discovery set (that is, the index is scaled to have zero mean and unit standard deviation in the discovery set).

## Results

### Study overview

Initially, we developed the optimal DNAme-based classifier to identify women with CIN3+ (i.e., the WID-CIN test). Then, to validate the *diagnostic* capacity of the WID-CIN test for CIN3+, we applied it to the diagnostic validation set to test the discrimination of CIN3+ and CIN2 against hrHPV-pos/Cyt-pos women with a histological diagnosis of CIN1 or hrHPV-pos/Cyt-neg women. Finally, to validate the *predictive* capacity of the WID-CIN test for CIN3+, we applied it to the predictive validation set to test the detection of hrHPV-pos/Cyt-neg women who develop CIN3+ in the future, as they should be targeted for closer surveillance in clinical practice.

### Development of the WID-CIN test

Previously, we found that methylation differences may vary due to immune cell type composition in cases compared to controls [31]. Hence, we assessed the level of cell type heterogeneity in each cervical cytology sample using EpiDISH [28], an algorithm that infers the relative proportion of epithelial cells, fibroblasts, and seven subtypes of immune cells in each sample. The cell type distributions were broadly similar between CIN3+ cases and controls with an increase in immune cells in CIN2 and CIN3+ cases (Additional file 1: Fig. S1).

When assessing the ~850,000 CpG sites included in the EPIC array [32], after false discovery rate adjustment, we found 158,434 CpGs to be significantly differentially methylated between CIN3+ cases and controls with the greatest differences in epithelial cells and with a skew towards hypermethylation in CIN3+ cases (Additional file 1: Fig. S1).

We used a machine learning technique called ridge regression to derive a diagnostic methylation signature to detect CIN3 or invasive cervical cancer, termed the WID-CIN test. It was developed solely using the discovery set and was subsequently applied to the independent validation sets. The discovery set (Table 1) consisted of 170 Cyt-pos samples (96% hrHPV-pos) at CIN3+ (164 CIN3/AIS and 6 invasive cancers) as cases and 202 hrHPV-pos/Cyt-neg samples as controls. We derived a diagnostic methylation signature to detect CIN3 or invasive cervical cancer, called the WID-CIN test. The WID-CIN test is based on a linear combination of the top 5000 differentially methylated CpGs (see Additional file 2). We found that CpGs selected for the WID-CIN test were enriched for Open Sea regions and depleted for CpG islands (Additional file 1: Fig. S1). Differential methylation at genes *FAM19A4*, *EPB41L3*, *PAX1*, and *SOX1* has previously been used to identify CIN2+ lesions [13], and these were represented by CpGs within the 5000 CpGs used to build the WID-CIN index.

### Validation of the diagnostic capacity of the WID-CIN test

**Fig. 1:**
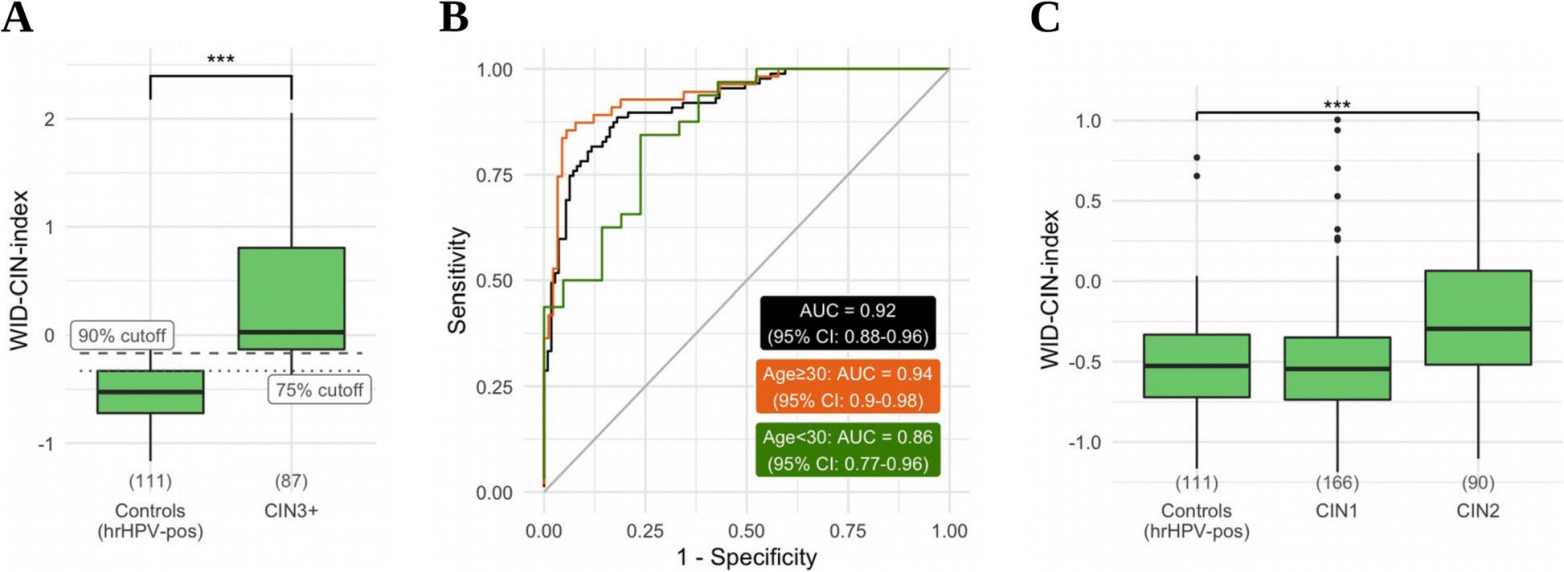
Distribution of the WID-CIN index in the diagnostic validation set (**A**). Receiver operating characteristic (ROC) curve corresponding to the diagnostic validation set with separate curves for women ≥ 30 years and < 30 years of age (**B**). Distribution of the WID-CIN index in CIN1 and CIN2 cases in the diagnostic validation set (**C**)

We then applied the WID-CIN test to the diagnostic validation set consisting of 87 CIN3+, 90 CIN2, and 166 CIN1 cases (98% hrHPV-pos) and 111 hrHPV-pos/Cyt-neg controls. For the CIN3+ cases and Cyt-neg controls, computing the WID-CIN index for each sample (Fig. 1A) resulted in an AUC of 0.92 (95% CI: 0.88–0.96) (Fig. 1B). Discriminatory performance was independent of immune cell proportion (Additional file 1: Fig. S2). The performance of the WID-CIN test was slightly better in ≥30-year-old women (AUC 0.94; 95% CI 0.90–0.98) compared to women <30 years (AUC 0.86; 95% CI 0.77–0.96) (Fig. 1B). At a specificity of 50%, almost all CIN3+ cases (96.6%) were correctly classified using the WID-CIN test (Fig. 1B). The WID-CIN index of Cyt-neg controls and Cyt-pos (CIN1) cases was almost identical (Fig. 1C).

At a specificity (CIN1 histology or normal cytology; ≤ CIN1) of 50%, 75%, and 90%, the WID-CIN test yielded a sensitivity of 96.6% (95% CI: 92.7–100), 89.7% (95% CI: 83.3–96.1), and 78.2% (95% CI: 69.5–86.8) for CIN3+ at all ages (Table 2). The respective sensitivities were even higher for women ≥30 years (Table 2). As expected, the performance of the WID-CIN test was lower in women <30 years; nevertheless, at a specificity of 75% (≤ CIN1), the sensitivity for CIN3+ was still 65.6% (95% CI: 49.2–82.1). We compared the WID-CIN index across different HPV subtypes (Additional file 1: Table S1) and found that the index was more elevated in samples with HPV16 (Additional file 1: Fig. S2).

**Table 2:** Sensitivity (detection of CIN2 or CIN3 and invasive cancers) of the WID-CIN test at different levels of specificity (CIN1 or normal cytology) and age groups in the diagnostic validation set. All three invasive cancers had a WID-CIN index value above the 90% specificity cutoff

	Age group	WID-CIN index cutoff	Sensitivity CIN2 (95% CI) [***N***/total]	Sensitivity CIN3+ (95% CI) [***N***/total]
**Specificity (≤CIN1) at 50%**	All ages	−0.53	75.6% (66.7–84.4) [68/90]	96.6% (92.7–100) [84/87]
	< 30 years	−0.56	70.6% (55.3–85.9) [24/34]	96.9% (90.8–100) [31/32]
	≥ 30 years	−0.49	73.2% (61.6–84.8) [41/56]	96.4% (91.4–100) [53/55]
**Specificity (≤CIN1) at 75%**	All ages	−0.34	54.4% (44.2–64.7) [49/90]	89.7% (83.3–96.1) [78/87]
	< 30 years	−0.17	35.3% (19.2–51.4) [12/34]	65.6% (49.2–82.1) [21/32]
	≥ 30 years	−0.33	55.4% (42.3–68.4) [31/56]	92.7% (85.9–99.6) [51/55]
**Specificity (≤CIN1) at 90%**	All ages	−0.16	38.9% (28.8–49) [35/90]	78.2% (69.5–86.8) [68/87]
	< 30 years	−0.06	23.5% (9.3–37.8) [8/34]	50% (32.7–67.3) [16/32]
	≥ 30 years	−0.21	44.6% (31.6–57.7) [25/56]	87.3% (78.5–96.1) [48/55]

We compared the performance of the WID-CIN test with those tests which are currently considered to be gold standard (i.e., PAP cytology [5]) or very promising candidates (i.e., dual staining cytology [5], the QIAsure™ Methylation Test which utilizes *FAM19A4/miR124-2* methylation [17], and other DNAme markers [13]) to triage hrHPV-pos women (Table 3). Although these studies are not directly comparable (see Table 3 legend), fixing the specificity of the WID-CIN test at 78%, which is the highest specificity among the other tests, the sensitivity of the WID-CIN test is 89.7% (95% CI: 83.3–96.1). The fact that the WID-CIN test is significantly better compared to the other tests is particularly impressive because 150/454 (33.0%) samples of our diagnostic validation set consisted of samples from women <30 years whereas almost all the data for the other tests in Table 3 were based on samples from women ≥30 years in which the performance is known to be substantially better.

**Table 3:** Specificity (CIN1 or normal cytology) and sensitivity (detection of CIN3 or invasive cancer) of specific strategies to triage hrHPV-positive women (95% confidence intervals)

	Specificity ≤ CIN1 (95% CI)	Sensitivity CIN3+ (95% CI)
**Cytology (PAP)** [5]	76.1% (74.6–77.7)	51.9% (45.4–58.3)
**Dual stain cytology (p16/Ki-67)** [5]	75.6% (74.0–77.1)	74.9% (69.0–80.2)
**QIAsure™ methylation test** [17]	78.3% (76.4–80.0)	78.6% (73.5–83.7)
**Methylation markers (pooled meta-analysis)**	75.9% (71.9–79.5)	70.5% (64.8–75.6)
**WID-CIN test**	78.0% (73.1–82.9)	89.7% (83.3–96.1)

The WID-CIN index has been fixed at 78% (based on the highest level by the other strategies) in order to make the sensitivity comparable. Wright et al. [5] included all women ≥25 years of age with valid cervical biopsy and cobas®HPV test results from the cross-sectional phase of the ATHENA study who were referred for colposcopy. Bonde et al. [17] conducted an EU-multicenter, retrospective study (samples collected at four European centers) to evaluate the clinical performance of the FAM19A4/miR124-2 methylation-based molecular triage test as a substitute or addition to cytology as reflex testing of HPV screen-positive women. Would we have excluded women < 29 years of age, the sensitivity of the WID-CIN test at a fixed specificity of 78% is 92.2%

### Validation of the predictive capacity of the WID-CIN test

**Fig. 2:**
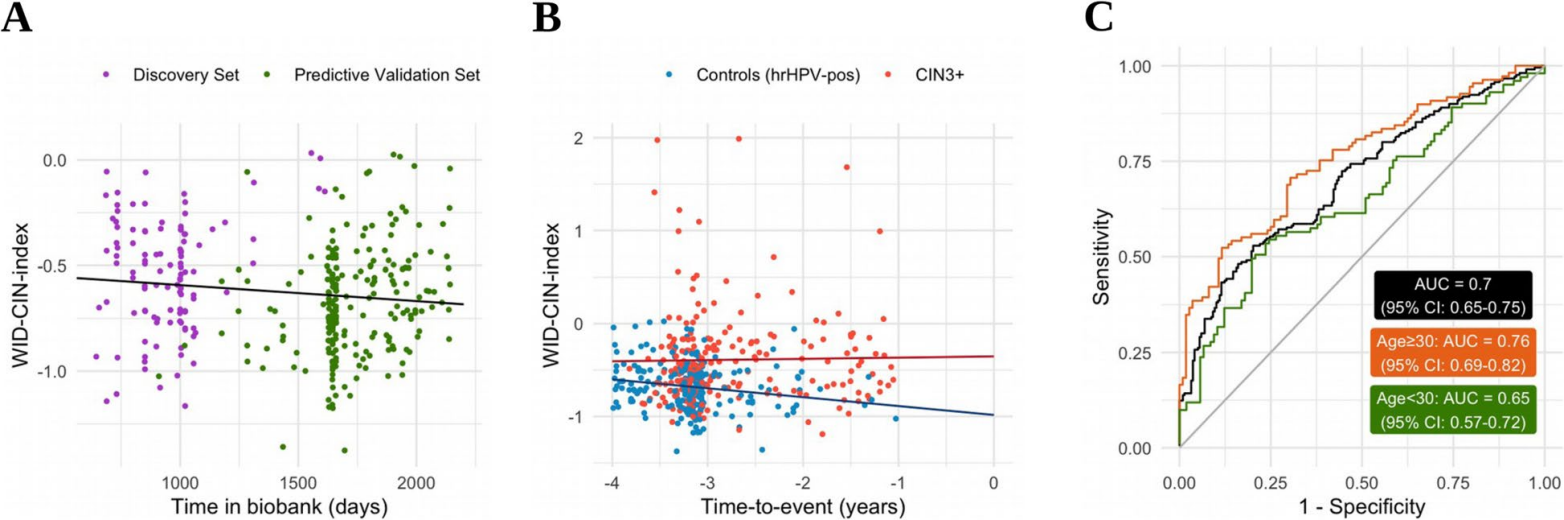
Dependence of the WID-CIN index in hrHPV-positive control samples on biobank storage time (**A**). The WID-CIN index in the predictive validation set consisting of hrHPV-positive and cytology-negative samples taken 1–4 years prior to either a diagnosis with CIN3+ (red points) or censoring (blue points) (**B**). ROC curve corresponding to the predictive validation set (**C**)

The validation set of predicting future CIN3+ development was comprised of 418 hrHPV-positive/Cytology-neg women and 10 hrHPV-negative/Cytology-neg women of whom 210 were diagnosed with CIN3+ 1 to 4 years after they provided their sample and 218 were disease-free within the same period (Table 1). Sample cell type composition was broadly comparable to the discovery set (Additional file 1: Fig. S3). As these samples had been stored longer in the biobank compared to those samples used for the discovery and diagnostic validation, the longer storage time significantly reduced the WID-CIN index (Fig. 2A; *p* = 0.044), making it impossible to apply the same WID-CIN index cutoffs as chosen in the previous set. Nevertheless, the WID-CIN index was elevated in a percentage of these Cyt-neg samples up to 4 years prior to the CIN3+ diagnosis (Fig. 2B) with an overall AUC of 0.70 (95% CI: 0.65–0.75) (Fig. 2C). The performance was better for women >30 years old (AUC 0.76; 95% CI 0.69–0.82) compared to women ≤30 years (AUC 0.63; 95% CI 0.57–0.72) (Fig. 2C).

At a specificity of 50%, 75%, and 90%, the WID-CIN test yielded a sensitivity of 74.3% (95% CI: 68.4–80.2), 54.8% (95% CI: 48.0–61.5), and 36.7% (95% CI: 30.1–43.2) to predict the future risk for CIN3+ at all ages (Table 4). The respective sensitivities were even higher for women ≥30 years. Again, as expected, the performance of the WID-CIN test was lower in women <30 years; nevertheless, at a specificity of 50%, the sensitivity for future CIN3+ was still 61.4% (95% CI: 51.9–70.9). A Kaplan-Meier plot suggests that the WID-CIN test effectively identifies women at risk of CIN3+ 2–4 years after sample acquisition (Additional file 1: Fig. S3).

**Table 4:** Sensitivity (detection of CIN3+) of the WID-CIN test at different levels of specificity and age groups in the predictive validation set

	Age group	WID-CIN index cutoff	Sensitivity CIN3+ (95% CI) [***N***/total]
**Specificity at 50%**	All ages	−0.70	74.3% (68.4–80.2) [156/210]
	< 30 years	−0.59	61.4% (51.9–70.9) [62/101]
	≥ 30 years	−0.75	80.7% (73.3–88.1) [88/109]
**Specificity at 75%**	All ages	−0.52	54.8% (48.0–61.5) [115/210]
	< 30 years	−0.45	53.5% (43.7–63.2) [54/101]
	≥ 30 years	−0.60	56.9% (47.6–66.2) [62/109]
**Specificity at 90%**	All ages	−0.33	36.7% (30.1–43.2) [77/210]
	< 30 years	−0.26	29.7% (20.8–38.6) [30/101]
	≥ 30 years	−0.46	42.2% (32.9–51.5) [46/109]

## Discussion

Cervical cancer screening is one of the foremost success stories in medicine in general, and oncology in particular. Here, we have provided evidence that an objective DNA methylation signature, the WID-CIN test, outperforms cytology as a tool to triage hrHPV-pos women for colposcopy referral. We have demonstrated that, in hrHPV-pos women, the WID-CIN test is able to (i) detect almost all (96.6%) prevalent CIN3+, while ruling out 50% of those who have no cytologic abnormality or a CIN1 on biopsy, and (ii) identify those hrHPV-pos/Cyt-neg women who will present with CIN3+ within 1–4 years. We note that the sensitivity for detection of CIN2 was lower than that for CIN3+ (73.2%). Given that a minority of CIN2 cases are estimated to eventually progress to CIN3+ (18%), in particular in women aged <30 (11%) [33], “overdiagnosis” of CIN2 may not always be beneficial.

Whereas a plethora of DNA methylation markers have been identified and assessed in cervical liquid-based cytology samples and deemed to be promising [13], only a small number of studies assessed the clinical validity of these markers in a screening setting. Using DNAme levels of a combination of two genes (i.e., *MAL* and *miR-124-2*), Verhoef et al. [34] demonstrated in a prospective clinical trial (albeit based on self-collected samples) that triaging HPV-pos women with DNA methylation provided a lower sensitivity (67.5%) compared to cytology-triaging (74.8%) and required almost twice as many colposcopy referrals. As this study was performed on women aged 33 years or older, the performance of these methylation markers would presumably have been substantially worse in younger women [15]. Although we also observed this age-dependent performance in the WID-CIN test, in young women (<30 years), we were able to achieve a sensitivity of 66% at a 75% specificity.

The comparison of the WID-CIN test with QIAsure, a commercially available DNAme test, shows that the WID-CIN shows a significantly improved performance. This is particularly impressive because almost all women in the QIAsure set were ≥30 years with a mean age of 40.7 years (all tests perform substantially better in older women) whereas the mean age in our set was 33.7 years.

We propose that the cellular heterogeneity of cervical liquid-based cytology samples is currently underappreciated, including at the level of human DNA which includes DNA from cell debris not visible at the microscopic level when assessing cytology [35]. We observed a high variability in the proportion of epithelial and immune cells in LBC samples, ranging from only epithelial cells without immune cells to samples that almost exclusively consisted of immune cells with few epithelial cells present. Importantly, we have thoroughly assessed and concluded that the WID-CIN test performance is independent of sample heterogeneity, which may suggest that it could perform equally well in self-collected samples, but this needs to be assessed in future studies.

The WID-CIN test exhibited high sensitivity and specificity across a variety of settings, although a lower AUC was observed in a diagnostic setting in women below 30 and in samples predating disease. The lower performance in women below 30 is in line with the performance of any other tests for cervical cancer screening (including cytology [36]) that also perform worse in this age group. The performance was also lower in as of yet disease-free women that developed CIN3+ up to 4 years after sample collection. Our observation that the WID-CIN test is able to identify HPV-pos women who show no abnormal cells in their cervical liquid-based cytology sample but develop CIN3+ between 1 and 4 years later may suggest that the WID-CIN test is not only reflective of an epigenetic cancer program, but in fact reflective of an individual predisposition to progress to a cervical (pre-) cancer upon infection with HPV. To test this hypothesis, samples from women prior to HPV infection will need to be analyzed to assess whether the WID-CIN test would have predicted the disease development even before the presence of the carcinogen. Nonetheless, the WID-CIN test does, as perhaps expected, have a higher diagnostic than predictive performance, as reflected by the higher AUC.

The strengths of this study include the use of only samples from a well-defined population-based screening cohort under careful design to control for potential bias due to factors such as age, sample year, and time of storage, with a comprehensive registry linkage strategy that enabled the identification of samples long preceding disease. In addition, we employed an epigenome-wide approach for identifying the most informative CpG sites to identify women with or at risk for CIN3+. Our limitations include that we sampled women with CIN3+ through screening programs only and did not include women with CIN3+ who presented (with symptoms) at gynecological or oncological units. However, we consider the generalizability advantage of this strategy to outweigh the potential drawbacks, since we aimed to identify a triage strategy suited for mass screening, which by definition will primarily consist of asymptomatic women.

## Conclusions

We have demonstrated the unprecedented performance of a comprehensive DNA methylation classifier — the WID-CIN test — in identifying hrHPV-pos women with or at future risk of CIN3+. The fact that the test principle (i.e., analysis of DNAme of a combination of CpGs on an array) not only identifies women with CIN3+ but also women with ovarian [37] and breast cancer [29] (WID-OC and WID-BC) suggests that the WID-CIN test could be rapidly prioritized for cost-effectiveness analyses and potential quick implementation in the clinical arena. In addition to array-based detection of CIN3+, in ongoing work, we have developed a multiplexed MethyLight PCR-based test, the WID-qCIN test, that amplifies regions in the genes *DPP6*, *RALYL*, and *GSX1* and exhibits excellent sensitivity and specificity in both diagnostic and predictive settings (Herzog, Sundström et al., submitted). Planned large-scale future studies prospectively evaluating the use of WID tests (either array- or PCR-based) side by side with the current standard of care will provide evidence of their performance in real-world settings.

## Supplementary Information

**Additional file 1. **Supplementary figures and tables:** Figure S1.** Cell-type composition in the combined Discovery Set and Diagnostic Validation Set as determined by the EpiDISH algorithm (A). Distribution of p-values after comparing hrHPV-positive controls to CIN3+ cases in the Discovery Set (based on a linear regression model with adjustment for age and immune cell proportion) (B). Distribution of the estimated epithelial and immune delta-betas (C). Performance of ridge and lasso classifiers based on out-of-bag estimates from 10-fold cross validation on the Discovery Set (D). Odds ratios when comparing the genomic annotation of the 5,000 CpGs comprising the WID-CIN-index to the 777,005 CpGs that were used in the analysis (E). **Figure S2.** Dependence of the WID-CIN-index on immune cell proportion in the hrHPV-positive controls and CIN3+ cases from the Diagnostic Validation Set (A). Dependence of the WID-CIN-index on age in the hrHPV-positive controls and CIN3+ cases from the Diagnostic Validation Set (B). The WID-CIN-index in different subgroups and HPV genotypes from the Diagnostic Validation Set (C). The other high risk genotype category consists of genotypes 31, 33, 35, 39, 45, 51, 52, 56, 58, 59, 66 and 68. Only samples that tested positive for one of the three genotype categories were included in the plot. **Figure S3.** The cell-type composition of samples from the predictive validation set based on the EpiDISH algorithm (A). Dependence of the WID-CIN-index on age in the hrHPV-positive controls and CIN3+ cases from the Predictive Validation Set (B). Kaplan-Meier curves from the Predictive Validation Set in which samples have been split into those below and above the 75% specificity cutoff (C). **Table S1.** Summary of hrHPV genotypes in the Discovery Set (A), Diagnostic Validation Set (B) and Predictive Validation Set (C). The other high risk genotype category consists of genotypes 31, 33, 35, 39, 45, 51, 52, 56, 58, 59, 66 and 68. Note that rows may not sum to the total sample number as some samples tested postive for multipe genotypes.

**Additional file 2.** The top 5,000 differentially methylated CpGs.

## Data Availability

DNAme data that support the findings of the study have been deposited in the European Genome-phenome Archive (EGA) database with the accession code EGAS00001005078 [38] (https://ega-archive.org/studies/EGAS00001005078). The authors declare that all other data supporting the findings of this study are available within the article and its supplementary files.

## Abbreviations

HPV: Human papillomavirus
hrHPV: High-risk human papillomavirus
Cyt: Cytology
CIN: Cervical intraepithelial neoplasia
CIN3+: Cervical intraepithelial neoplasia grade 3 or higher
Cyt-pos/neg: Cytology positive/negative
hrHPV-pos/neg: High-risk human papillomavirus positive/negative
WID-CIN: Women’s cancer risk IDentification-Cervical Intraepithelial Neoplasia
AIS: Adenocarcinoma in situ
DMP: Differentially methylated position
IC: Immune cell
LBC: Liquid-based cytology
