# Supplementary material for: The WID-CIN test identifies women with, and at risk of, cervical intraepithelial neoplasia grade 3 and invasive cervical cancer
Source: Genome Med. 2022 Oct 19;14:116. doi: 10.1186/s13073-022-01116-9 (PMC9580141; doi:10.1186/s13073-022-01116-9)
Supplement: Supplementary file 1 — Additional file 1. Supplementary figures and tables: Figure S1. Cell-type composition in the combined Discovery Set and Diagnostic Validation Set as determined by the EpiDISH algorithm (A). Distribution of p-values after comparing hrHPV-positive controls to CIN3+ cases in the Discovery Set (based on a linear regression model with adjustment for age and immune cell proportion) (B). Distribution of the estimated epithelial and immune delta-betas (C). Performance of ridge and lasso classifiers based on out-of-bag estimates from 10-fold cross validation on the Discovery Set (D). Odds ratios when comparing the genomic annotation of the 5,000 CpGs comprising the WID-CIN-index to the 777,005 CpGs that were used in the analysis (E). Figure S2. Dependence of the WID-CIN-index on immune cell proportion in the hrHPV-positive controls and CIN3+ cases from the Diagnostic Validation Set (A). Dependence of the WID-CIN-index on age in the hrHPV-positive controls and CIN3+ cases from the Diagnostic Validation Set (B). The WID-CIN-index in different subgroups and HPV genotypes from the Diagnostic Validation Set (C). The other high risk genotype category consists of genotypes 31, 33, 35, 39, 45, 51, 52, 56, 58, 59, 66 and 68. Only samples that tested positive for one of the three genotype categories were included in the plot. Figure S3. The cell-type composition of samples from the predictive validation set based on the EpiDISH algorithm (A). Dependence of the WID-CIN-index on age in the hrHPV-positive controls and CIN3+ cases from the Predictive Validation Set (B). Kaplan-Meier curves from the Predictive Validation Set in which samples have been split into those below and above the 75% specificity cutoff (C). Table S1. Summary of hrHPV genotypes in the Discovery Set (A), Diagnostic Validation Set (B) and Predictive Validation Set (C). The other high risk genotype category consists of genotypes 31, 33, 35, 39, 45, 51, 52, 56, 58, 59, 66 and 68. Note that rows may not sum to the total [file 13073_2022_1116_MOESM1_ESM.pdf]

ADDITIONAL FILE 1

The WID-CIN test identifies women with, and at risk of, cervical intraepithelial neoplasia grade 3 and invasive cervical cancer

James E. Barrett, Karin Sundström, Allison Jones, Iona Evans, Jiangrong Wang, Chiara Herzog, Joakim Dillner, Martin Widschwendter

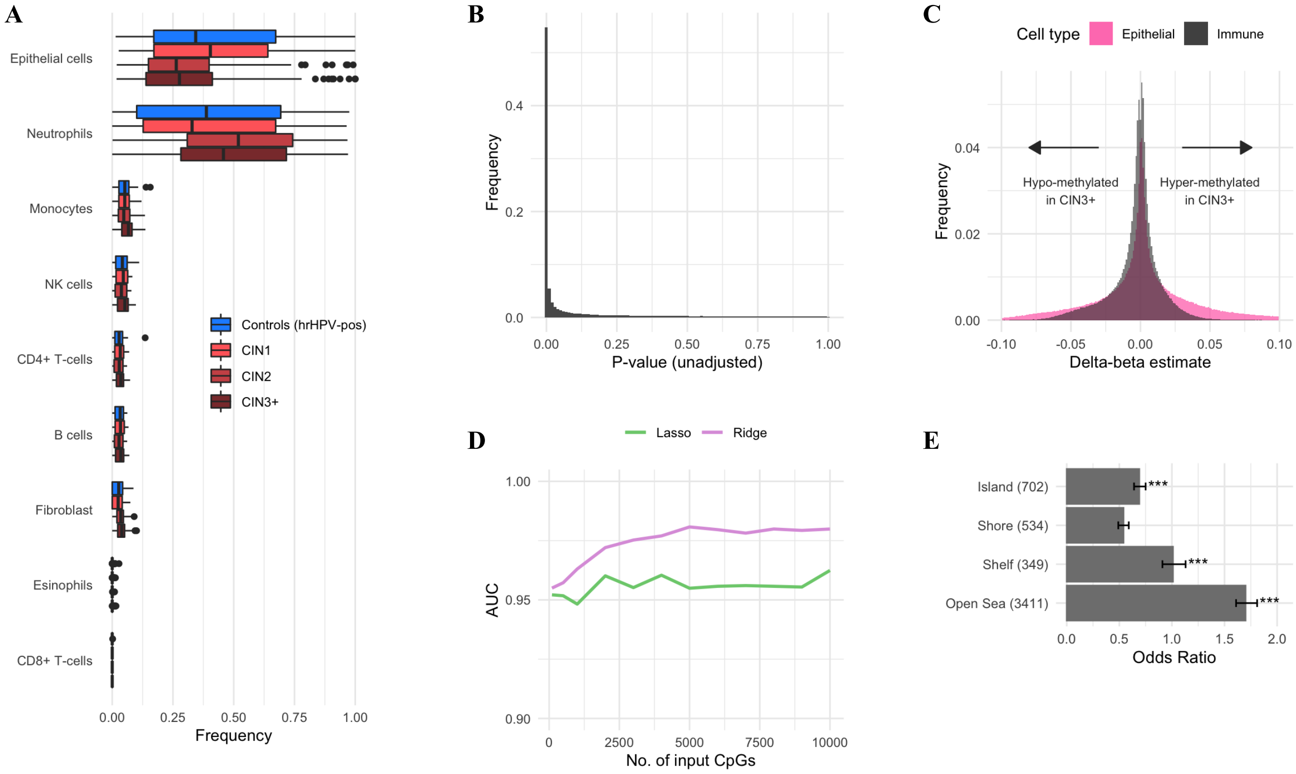

Figure S1. Cell-type composition in the combined Discovery Set and Diagnostic Validation Set as determined by the EpiDISH algorithm (A). Distribution of p-values after comparing hrHPV-positive controls to CIN3+ cases in the Discovery Set (based on a linear regression model with adjustment for age and immune cell proportion) (B). Distribution of the estimated epithelial and immune delta-betas (C). Performance of ridge and lasso classifiers based on out-of-bag estimates from 10-fold cross validation on the Discovery Set (D). Odds ratios when comparing the genomic annotation of the 5,000 CpGs comprising the WID-CIN-index to the 777,005 CpGs that were used in the analysis (E).

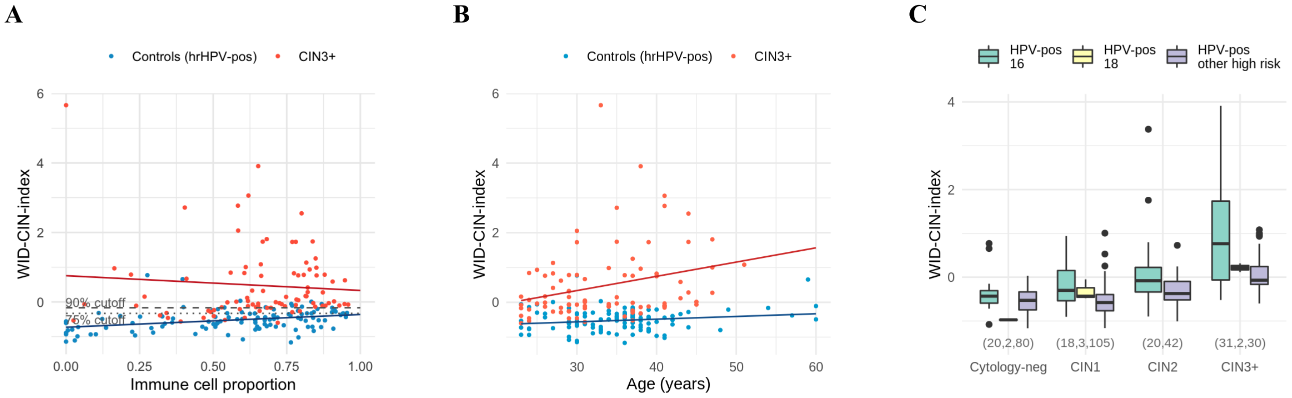

Figure S2. Dependence of the WID-CIN-index on immune cell proportion in the hrHPV-positive controls and CIN3+ cases from the Diagnostic Validation Set (A). Dependence of the WID-CIN-index on age in the hrHPV-positive controls and CIN3+ cases from the Diagnostic Validation Set (B). The WID-CIN-index in different subgroups and HPV genotypes from the Diagnostic Validation Set (C). The other high risk genotype category consists of genotypes 31, 33, 35, 39, 45, 51, 52, 56, 58, 59, 66 and 68. Only samples that tested positive for one of the three genotype categories were included in the plot.

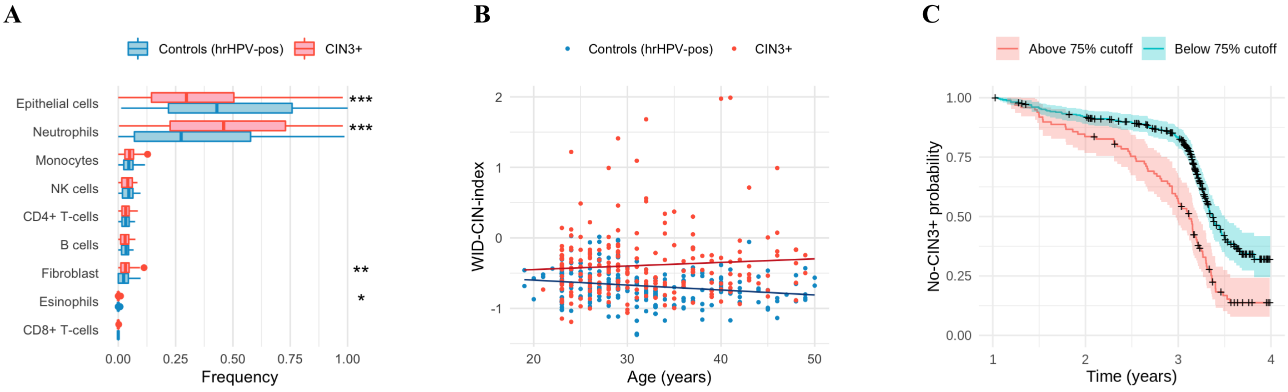

Figure S3. The cell-type composition of samples from the predictive validation set based on the EpiDISH algorithm (A). Dependence of the WID-CIN-index on age in the hrHPV-positive controls and CIN3+ cases from the Predictive Validation Set (B). Kaplan-Meier curves from the Predictive Validation Set in which samples have been split into those below and above the 75% specificity cutoff (C).

A) Discovery Set

Pathological diagnosis	hrHPV-pos 16	hrHPV-pos 18	hrHPV-pos (other high risk)	hrHPV-neg	Unknown
Invasive cancer (n=6)	1	4	3	0	0
AIS (n=7)	4	2	3	0	0
CIN3 (n=157)	92	18	101	5	1
Cytology-neg (n=202)	35	21	167	0	0

B) Diagnostic Validation Set

Pathological diagnosis	hrHPV-pos 16	hrHPV-pos 18	hrHPV-pos (other high risk)	hrHPV-neg	Unknown
Invasive cancer (n=3)	3	0	1	0	0
CIN3 (n=84)	48	7	52	1	0
CIN2 (n=90)	42	8	68	1	0
CIN1 (n=166)	41	18	142	3	0
Cytology-neg (n=111)	26	4	88	0	1

C) Predictive Validation Set

Pathological diagnosis	hrHPV-pos 16	hrHPV-pos 18	hrHPV-pos (other high risk)	hrHPV-neg	Unknown
CIN3+ 1-4 years later (n=210)	93	22	136	10	2
No CIN3+ 1-4 year later (n=218)	38	18	179	0	3

Table S1. Summary of hrHPV genotypes in the Discovery Set (A), Diagnostic Validation Set (B) and Predictive Validation Set (C). The other high risk genotype category consists of genotypes 31, 33, 35, 39, 45, 51, 52, 56, 58, 59, 66 and 68. Note that rows may not sum to the total sample number as some samples tested postive for multipe genotypes.
